# Interoccurrence time statistics in fully-developed turbulence

**DOI:** 10.1038/srep27452

**Published:** 2016-06-10

**Authors:** Pouya Manshour, Mehrnaz Anvari, Nico Reinke, Muhammad Sahim, M. Reza Rahimi Tabar

**Affiliations:** 1 Department of Physics, Faculty of Sciences, Persian Gulf University, Bushehr 75169, Iran; 2 Institute of Physics and for wind, Carl von Ossietzky University of Oldenburg, 26111 Oldenburg, Germany; 3 Mork Family Department of Chemical Engineering Materials Science, University of Southern California, Los Angeles, California 90089-1211, USA; 4 Department of Physics, Sharif University of Technology, Tehran 11155-9161, Iran

## Abstract

Emergent extreme events are a key characteristic of complex dynamical systems. The main tool for detailed and deep understanding of their stochastic dynamics is the statistics of time intervals of extreme events. Analyzing extensive experimental data, we demonstrate that for the velocity time series of fully-developed turbulent flows, generated by (i) a regular grid; (ii) a cylinder; (iii) a free jet of helium, and (iv) a free jet of air with the Taylor Reynolds numbers Re_*λ*_ from 166 to 893, the interoccurrence time distributions *P*(*τ*) above a positive threshold *Q* in the inertial range is described by a universal *q*- exponential function, *P*(*τ*) = *β*(2 − *q*)[1 − *β*(1 − *q*)*τ*]^1/(1−*q*)^, which may be due to the *superstatistical* nature of the occurrence of extreme events. Our analysis provides a *universal* description of extreme events in turbulent flows.

Dynamical systems in biology, physics, chemistry, computer science, social and other types of networks, and many other fields and phenomena exhibit rich and complex behavior that are not amenable to the classical methods of analysis. Although there is no consensus on what constitutes complex systems, they are usually referred to as such because they typically consist of many subparts that interact with each other over many length and/or time scales, not uniquely but in several ways. Due to their complexity, the difficulty in precisely describing their behavior and properties, and the ubiquity of complex dynamical systems in nature, one approach to the analysis of such systems has been based on trying to identify their *universal* features. That is, one tries to discover whether there are certain features of complex dynamical systems that are exhibited by a large class of them, regardless of the details of their structure. If such universal features can be identified, then one can utilize them to develop the simplest model of such systems that exhibit the universal properties and utilize it to gain further insights into their dynamics.

One of the main features of experimental data for complex dynamical systems is their small time scale intermittency. Intuitively, intermittency represents the strongly correlated fluctuations that lead to deviations of increments probability distribution functions (PDF) from Gaussian statistics at small scales and is defined by, *δx*_*r*_ = *x*(*t* + *r*) − *x*(*t*), for a given small *r*. The stretched exponential like behavior of *P*(*δx*_*r*_) changes as the scale *r* increases from the smallest to the largest ones.

A prototype of complex dynamical systems is turbulent flow. As is well understood, any flow field is the result of the competition between two factors, namely, the inertial and viscous forces^1^. The Taylor Reynolds numbers is defined as *Re*_*λ*_ = *U*_rms_*λ*/*ν*, where *U*_rms_, *λ*, and *ν* are, respectively, the root mean-square of the fluctuations in the velocity field, the Taylor length scale of the flow field, and the kinematic viscosity^2^. In turbulent flows, which correspond to large *Re*_*λ*_, i.e. the dominance of inertial over viscous forces, the fluid particles follow complex trajectories resembling stochastic motion. The flow is characterized by an energy flux cascade from a large integral scale *L* towards the intermediate and eventually smallest scales in which energy is dissipated by molecular viscosity. The inertial range is defined by the scales *λ* < *l* < *L*.

Traditionally, the universality in fully-developed turbulence is expressed in terms of the multiscaling exponents that describe the multiscaling of the positive ordered structure functions of the velocity increments in the inertial range under the steady state condition. More precisely, the longitudinal velocity increments, 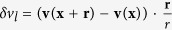, across a distance *r* (in the inertial range) is believed to behave as, 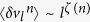, where *ζ*(*n*) is the scaling exponent. Here, the field **v**(**x**) is the velocity at location **x**. The Kolmogorov’s K41 theory^3^ predicts that, *ζ*(*n*) = *n*/3, but various experimental as well as numerical studies have indicated significant deviations of *ζ*(*n*) from *n*/3. Such deviations are referred to as *anomalous scaling*, and are due to the small-scale intermittent nature of fully-developed turbulence^1^. Many phenomenological models have been proposed^4^,^5^,^6^,^7^,^8^,^9^ to study the anomalous scaling.

Turbulent flow is also a system in which the motion of the fluid particles manifests complex fluctuations and possesses many universal features^10^,^11^,^12^,^13^,^14^,^15^. For example, the anomalous scaling exponents *ζ*(*n*) display^16^,^17^,^18^ universality with respect to the boundary conditions and the large-scale forcing mechanisms of fluid flow. Understanding how the complexity of turbulent flow, as a strongly complex non-equilibrium phenomenon with nonlinear memory, can be described by universal laws has been a great scientific challenge for decades.

On the other hand to gain deeper understanding of the stochastic dynamics of the velocity time series, one can study the statistics of time intervals of extreme events. It has been shown in recent years^19^,^20^,^21^,^22^,^23^,^24^,^25^,^26^,^27^,^28^,^29^,^30^,^31^,^32^ that the analysis of the interoccurrence time (IOT) series is a powerful tool for characterizing temporal properties of extreme events, the main cause of complexity in such systems. Let us introduces a positive/negative threshold *Q* and search for possible information inherent in the statistics of the time intervals *τ*_*i*_ between successive events above/below the threshold, as shown in Fig. 1, in order to identify the laws that govern the occurrence of the extreme events. In other words, the IOT series represent the reoccurrence of events that exceed a certain threshold or level *Q*, such that for larger values of *Q* the events are rare or extreme^28^,^29^,^30^. For uncorrelated processes, the PDF of *τ*_*i*_ is exponential, 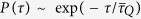, where 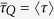 is the mean IOT for a given threshold *Q*, and the corresponding IOT sequence is also uncorrelated.

An empirical study indicated^31^,^33^ that, for a fixed mean time interval 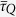 and a negative threshold *Q*, the distribution *P*(*τ*) of the IOT between the losses in a financial market below *Q* is described, for large ranges of time scales, by a universal *q*-exponentials given by,


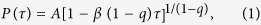


and the IOTs *τ*_*i*_ are also long-range correlated^32^. Here, *A* = *β*(2 − *q*) is a normalization factor. The question that arises is whether Eq. (1) is more general, and can describe extreme events in other complex dynamical systems.

In this paper we analyze the statistics of extreme events of fully-developed turbulent flows over a wide range of Reynolds numbers with distinct mechanisms of generation of turbulence, by analyzing the IOTs above a positive threshold *Q*. We analyze extensive experimental data for the velocity time series of turbulent flows generated by, (i) a regular grid; (ii) a cylinder; (iii) a free jet in helium^34^, and (iv) a free jet in air with Reynolds numbers Re_*λ*_ ranging from 166 to 893. We compute the statistics of the IOTs *τ*_*i*_ of the normalised velocity time series *v*(*t*) above a positive threshold *Q*. For given *Q*, the IOTs have mean and variance 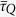 and *σ*_*Q*_, respectively. We demonstrate that the temporal occurrence of the extreme events of turbulent flows is characterized by a universal function over a wide range of *τ* that belong to the inertial range. We find, for *τ* values that are longer than the integral scale, that the IOT statistics has nonuniversal exponential type behaviour. We also show that the *q*-exponential form of the PDF of the IOT statistics may be due to the superstatistical nature of the occurrence of extreme events.

## Results

**Figure 2:**
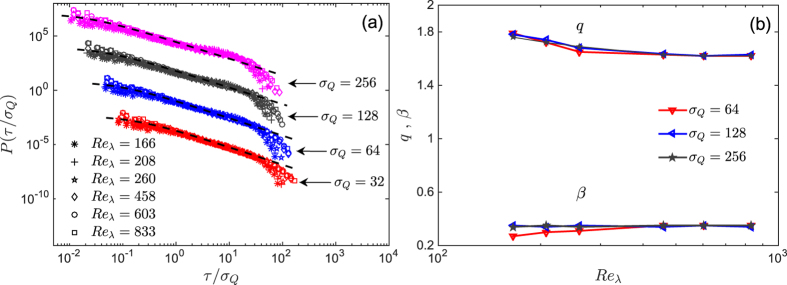
(**a**) The PDF *P*(*τ*/*σ*_*Q*_) of the IOTs for the normalized velocity time series for six Reynolds numbers Re*λ*. (**b**) Dependence of the parameters *q* and *b* on the Reynolds number. For large Re_*λ*_ they approach *β* ≈ 0.35 and *q* ≈ 1.62. The associated *q*-exponentials, Eq. (1), with the asymptotic values of *q* and *β* are shown by dashed lines in (**a**).

In Fig. 2(a) we present the computed IOT distributions *P*(*τ*) corresponding to the velocity time series *v*(*t*), measured for six Reynolds numbers and calculated for various *σ*_*Q*_ (or various thresholds *Q*). The plots are in logarithmic scales and have been shifted for better clarity. All the curves display a universal behavior in the inertial range in which the range increases for large Re_*λ*_. The inertial range in terms of *τ*/*σ*_*Q*_, such as for example for *σ*_*Q*_ = 32, is the interval 0.8–37. To determine the inertial range in each time series we calculate the Taylor and integral time scales. Such a behavior in the inertial range is modeled by the *q*-exponentials of Eq. (1). The dashed lines in Fig. 2(a) indicate *q*-exponentials function with the parameters, *β* ≈ 0.35, *q* ≈ 1.62, and 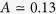. The dependence on the Reynolds number of *β* and *q* are shown in Fig. 2(b) for three *σ*_*Q*_. Clearly, for large Reynolds numbers *q* and *β* approach their universal values.

**Figure 3:**
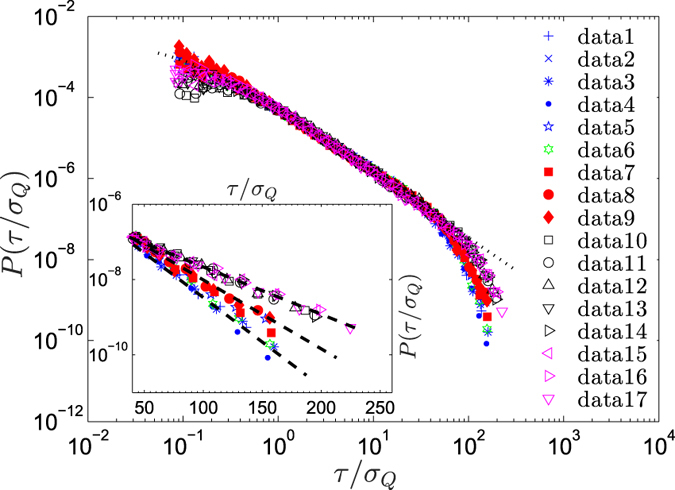
The PDF *P*(*τ*/*σ*_*Q*_) of the IOTs for the normalized velocity time series for several sets of data. Dotted line shows the *q*-exponential function, Eq. (1), with *β* ≈ 0.35 and *q* ≈ 1.62. The inertial range in terms of *τ*/*σ*_*Q*_ with *σ*_*Q*_ = 32 is the interval 0.8–37. The PDFs of the large IOTs, which are outside the inertial range, are shown in a semi-logarithmic plot in the inset in which the linear dashed lines indicate the nonuniversal exponential decay.

To check the generality of such universal behavior, we analyzed the velocity time series of various high-Reynolds number turbulent flows, generated by various mechanisms, and calculated their corresponding IOT distributions *P*(*τ*). Figure 3 demonstrates the IOT distributions of 17 normalized turbulent velocity series for several Reynolds number, all of which collapse onto the same universal curve in the inertial range given by Eq. (1) with the same parameters as in Fig. 2 (dotted line). We, therefore, conclude that the extreme events statistics of turbulent flows are very well characterized by a universal *q*-exponential function for the *τ*s that belong to the inertial range. Note that, as shown in the inset of Fig. 3, for the *τ*s that are outside of the inertial range, the PDFs of the IOT approach a simple nonuniversal exponential function. For small *τ*, on the other hand, the structure of the PDFs is caused by Taylor time scale.

**Figure 4:**
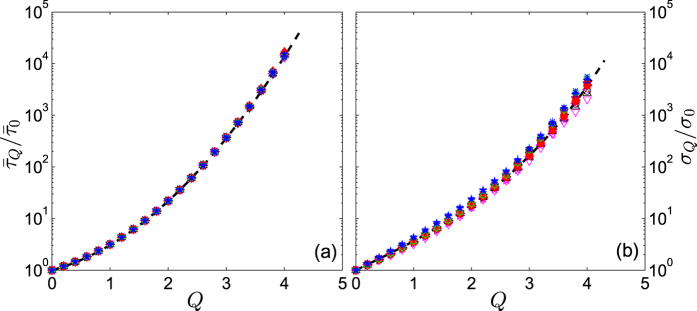
The *Q* dependence of (**a**) 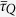, and (**b**) *σ*_*Q*_ of the normalized velocity time series and several Reynolds numbers. The dashed lines in (**a**,**b**) indicate the functional form, 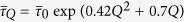 and *σ*_*Q*_ = *σ*_0_ exp(0.05*Q*^3^ + 1.25*Q*), respectively. Symbols are the same as in Fig. 3.

It should also be noted that, for each time series, there is a one-to-one correspondence between *Q* and 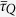 or *σ*_*Q*_. We present in Fig. 4 the dependence on the threshold *Q* of 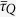 and *σ*_*Q*_ for the normalized velocity series in several turbulent flows. All the curves can be represented by, for example, the functional forms 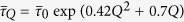 and *σ*_*Q*_ = *σ*_0_ exp (0.05*Q*^3^ + 1.25*Q*), where 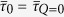 and *σ*_0_ = *σ*_*Q*=0_.

**Figure 5:**
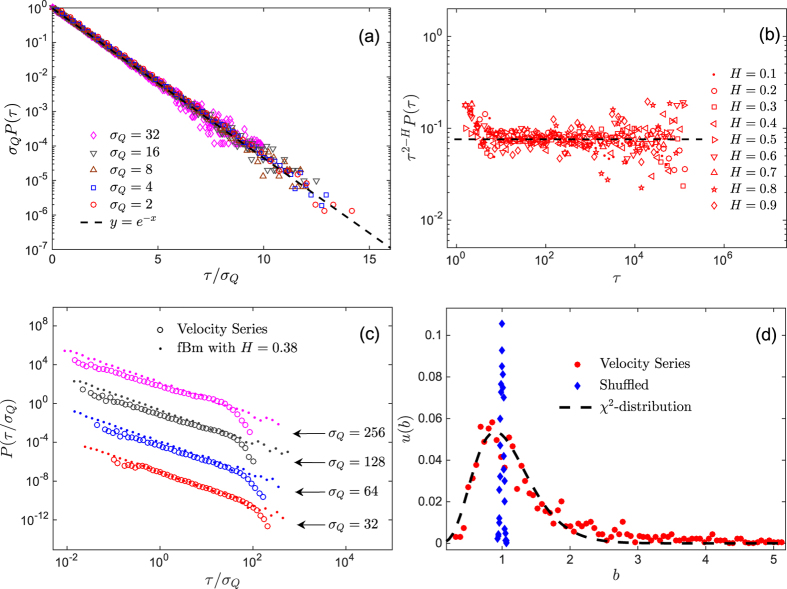
(**a**) The PDF of the normalized IOTs for the shuffled velocity time series with Re_*λ*_ = 603, and several standard deviations *σ*_*Q*_. All the data collapse onto the same exponential function, 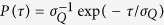. (**b**) The rescaled PDFs of the IOTs extracted from the fBm processes with various Hurst exponents, indicating a power-law form, 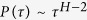. (**c**) The PDFs of the IOT of the velocity time series with Re_*λ*_ = 603, and that of a fBm process with *H* = 0.38, for several standard deviations. (**d**) The probability density *u*(*b*) for the normalized IOT sequences associated with the normalised velocity time series for Re_*λ*_ = 603 (circles) and its corresponding shuffled series (diamonds). Dashed line indicates the fitted *χ*^2^ distribution, Eq. (3), with a degree of freedom *k* ≃ 12.

To check the effect of the correlation structure of the velocity time series on the IOT statistics, we shuffled the velocity time series and calculated the PDF of the resulting series. The results are shown in Fig. 5(a) for a Reynolds number of 603 and five values of *σ*_*Q*_. All the curves collapse onto the same exponential function, 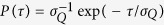. We note further that, the heavy tails of the PDF have no effect on the IOT statistics, since the time intervals between extreme events are studied, and the IOT statistics depend solely on the positions of such events.

To investigate the influence of the nonlinear nature of the velocity time series on PDFs of the IOT, we used the fractional Brownian motion (fBm), a centered self-affine Gaussian process that possesses linear long memory and is characterized by the Hurst exponent^35^ *H* ∈ (0, 1). *H* is defined by, 〈[*x*(*t* + *l*) − *x*(*t*)]^2^〉 = *c*_*H*_*l*^2*H*^, where *c*_*H*_ is a *H*-dependent constant. The fBm was introduced by Kolmogorov^36^ in connection with his work related to turbulence, and studied extensively later on by Mandelbrot and van Ness^37^. We note that the velocity time series in turbulence has nonlinear exponents *ζ*(*n*) with respect to *n*, while fBM has linear n-dependent exponents.

Then, we generated several fBM series with various Hurst exponents, and computed the PDFs of the corresponding IOTs. In Fig. 5(b) we present a plot of *τ*^2−*H*^*P*(*τ*) versus *τ* for various Hurst exponents. All the curves collapse onto a straight horizontal line, indicating a power-law behavior of the form, 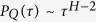, for the PDFs of the IOT of the fBM processes. Figure 5(c) presents the PDF of the IOT of the normalized velocity series for a Reynolds number of 603 and its linear corresponding fBM series with Hurst exponent of *H* ≈ 0.38, computed by detrended fluctuation analysis^38^. They are compared with synthetic fBM process with several *σ*_*Q*_. We can conclude that, the existence of any difference between the PDF of the IOT of the velocity time series and that of the fBM series can be due to the presence of the nonlinear correlation effects and influence of characteristic time scales *λ* and *L* in velocity time series.

To connect the parameters of the IOT statistics that we identify in this paper to the Kolmogorov exponent *ζ*(2), we note that in the limit 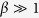 the IOT statistics is written as a power law, 
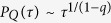
. On the other hand, in the linear approximation we also showed that 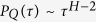. Thus, equating the two expressions yields, *q* = (3 − *H*)/(2 − *H*). The estimate, *q* ≈ 1.62 then yields, *H* ≈ 0.38, in complete agreement with the estimate reported for isotropic and homogeneous turbulence^39^. Note also that, the Hurst exponent is related to *ζ*(*n*) by *H* = *ζ*(*n* = 2)/2, hence establishing a direct relation between the Kolmogorov exponent and parameter *q* in the *q*-exponential function.

Further insight is gained by recognizing, as pointed out earlier, that the IOT distribution associated with an uncorrelated process is a simple exponential function, *P*(*τ*) ~ exp(−*bτ*), with *b* = 1/*σ*_*Q*_. Most IOTs are very sharp for large values of *b* (*σ*_*Q*_), whereas small values of *b* correspond to frequent occurrence of long IOTs. The existence of correlations in the process makes *b* a random variable with a probability density *u*(*b*). Then^40^,


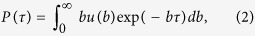


where the multiplier *b* in the integrand is a normalization factor. It is then clear that any deviation of *u*(*b*) from a delta function yields a nonexponential *P*(*τ*). It has been shown^41^,^42^ that if *b* is *χ*^2^-distributed, one obtains the *q*-exponential form of *P*(*τ*). Simply put, if {*x*_*i*_} represents a set of independent Gaussian variables *x*_*i*_, then 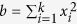 is distributed according to the *χ*^2^ distribution with *k* degrees of freedom, and


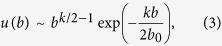


with *b*_0_ being a constant. To compute the distribution of *b*, one divides the *normalized* IOT sequences into a large number of equal windows with a size larger than the integral scale, estimates *b* = 1/*σ*_*Q*_ for each window, and constructs their distribution.

Thus, we constructed *u*(*b*) for the velocity times series with Re_*λ*_ = 603, as well as for its shuffled series. In Fig. 5(d) we present *u*(*b*) for the two series, along with their fit to the *χ*^2^ distribution. The velocity time series is well-represented by the *χ*^2^ distribution with *k* = 12, whereas its shuffled series tends to a delta function corresponding to *k* → ∞. Thus, the *q*-exponential form of the PDF of the IOT statistics may be due to the *superstatistical* nature of the occurrence of extreme events.

## Summary

It is well-known that the dynamics of many complex systems is characterized by the same universal principles, including high affinity for self-organization, pattern formation and synchronization that often generate extreme events. Turbulence is a prototype of such complex systems, as it possesses strong extreme events over small length (time) scales. Through extensive analyzes of the time series for fully-developed turbulent flows over a broad range of the Reynolds number and generated by various mechanisms, we have shown that above a positive threshold *Q* the probability distributions of the interoccurrence times of the velocity time series in the inertial range are well characterized and represented by the same (universal) *q*-exponentials function, *P*_*Q*_(*τ*) = *β*(2 − *q*)[1 + *β*(*q* − 1)*τ*]^1/(1−*q*)^, with *β* ≈ 0.35 and *q* ≈ 1.62. We also showed that the *q*-exponential form of the PDFs of the IOT may be due to the superstatistical nature of the occurrence of extreme events. Due to large relative distances between the sources of generating turbulence and the points of measurements, it was assumed in our analysis that turbulence is isotropic. A study of the role of anisotropy in statistical properties of the IOT series and study the relation between the exponents *ζ*(*n*) and the IOT of the velocity increments will be the next step in this research direction.

We believe that our method of analysis is applicable to other complex systems and phenomena in which extreme events occur, and yields valuable information for risk analysis and understanding of such events in complex dynamical systems^43^.

### Experimental Methodology

Large data sets of turbulent flows with Taylor Reynolds number Re_*λ*_ ranging from 166 to 893 were analyzed. The turbulent flows were generated by various mechanisms. The data correspond to four subclasses of data for which turbulence was generated by, (i) a regular grid^44^; (ii) a cylinder^44^; (iii) a free jet of low-temperature helium^45^, and (iv) a free jet of air^46^.

In the cases of (i) and (ii), measurements with a regular grid and in a cylinder’s wake were carried in the wind tunnel of the University of Erlangen, Germany^44^. The regular grid has a mesh width of size *M* = 0.05 m. The wake data were recorded at a downstream position of *x* = 32 m. The data generated by the cylinder’s wake were recorded at a downstream position of *x* = 40*D*, with the cylinder’s diameters being *D* = 0.02 m and 0.05 m.

In the (iii) case helium free-jet experiments were performed at CNRS, Grenoble, France^45^. They were carried out under cryogenic conditions in which the helium has very low viscosity and its free jet reaches high Reynolds numbers. The distance between the nozzle and the position of the measurement was *x* = 40*N*, where the nozzle’s diameter was *N* = 0.002 m.

In the (iv) case, the free-jet data in air were measured with two experimental setups at the University of Oldenburg, Germany. The first setup used was a closed chamber (1 × 1 × 2 m^3^ corresponding to width × width × downstreamlength) in which air was injected by means of a nozzle with a diameter of *N* = 0.008 m. The data for the free-jet turbulence were measured at *x* = 40 *N*. A second set of experiments was carried out with a freely-injected jet in the laboratory, where the distance between the free jet and the walls was on the order of 2 m. Therefore, the free jet stayed centered downstream and the measurements exhibited no bending of the jet. Measurements were carried out at *x* = 40 *N*, 60 *N* and 80 *N*, where the nozzle’s outlet was *N* = 0.1 m.

For all the measurements, the isotropy of turbulence was achieved due to the large relative distances between the points of generating turbulence and the points at which the measurements were carried out. Each dataset contained 1.25 × 10^6^ to 40 × 10^6^ samples of the longitudinal velocity. Almost all the measurements were performed with the hot-wire technique (mostly single wire), except^45^ those in which a microstructured hot-point probe was used. It is worth pointing out that all the experiments and the corresponding measurements were performed in such a way that the corresponding datasets contained high-quality data in terms of, e.g. the downstream position, the probe size, the length of the data log, and the sampling frequency. More information on the experiments and the datasets are given in the cited references. Table 1 lists the Reynolds numbers for the data sets used in Fig. 3.

## Additional Information

**How to cite this article**: Manshour, P. *et al*. Interoccurrence time statistics in fully-developed turbulence. *Sci. Rep*. **6**, 27452; doi: 10.1038/srep27452 (2016).

## Figures and Tables

**Figure 1:**
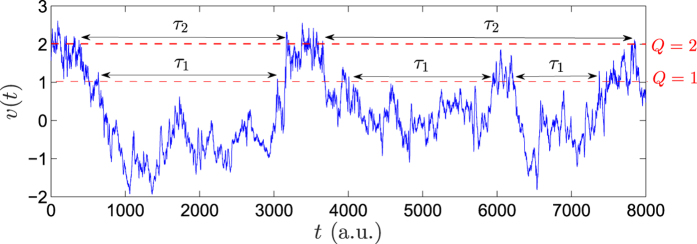
Schematic illustration of normalised velocity {*v*(*t*) → [*v*(*t*) − *ν*]/*σ*} interoccurrence times, where *sigma* is the standard deviation of the velocity time series. Shown are the values of *τ*_1_ and *τ*_2_ for the threshold *Q* = 1 (*σ*) and *Q* = 2 (*σ*) and for turbulent flow with Re_*λ*_ = 603. For given *Q*, values of *τ* have mean and variance 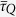 and *σ*_*Q*_, respectively.

**Table 1:** The data sets and their corresponding Reynolds numbers Re_*λ*_.

Data set	1	2	3	4	5	6	7	8	9	10	11	12	13	14	15	16	17
Re_*λ*_	534	584	653	750	893	463	458	603	833	602	690	755	774	864	456	581	467
