# Corrigendum: Interoccurrence time statistics in fully-developed turbulence

**DOI:** 10.1038/srep31937

**Published:** 2016-09-01

**Authors:** Pouya Manshour, Mehrnaz Anvari, Nico Reinke, Muhammad Sahimi, M. Reza Rahimi Tabar

Scientific Reports 6: Article number: 27452; 10.1038/srep27452 published online: 06 10 2016; updated: 09 01 2016.

The original version of this Article contained a typographical error in the spelling of the author Muhammad Sahimi, which was incorrectly given as Muhammad Sahim. This has now been corrected in the PDF and HTML versions of the Article.

